# Supplementary material for: Correlating Josephson supercurrents and Shiba states in quantum spins unconventionally coupled to superconductors
Source: Nat Commun. 2021 Feb 17;12:1108. doi: 10.1038/s41467-021-21347-5 (PMC7889868; doi:10.1038/s41467-021-21347-5)
Supplement: Supplementary file 1 — Supplementary Information [file 41467_2021_21347_MOESM1_ESM.pdf]

Supplementary information: Correlating Josephson supercurrents and Shiba states in quantum spins unconventionally coupled to superconductors

Felix Küster *et al.*

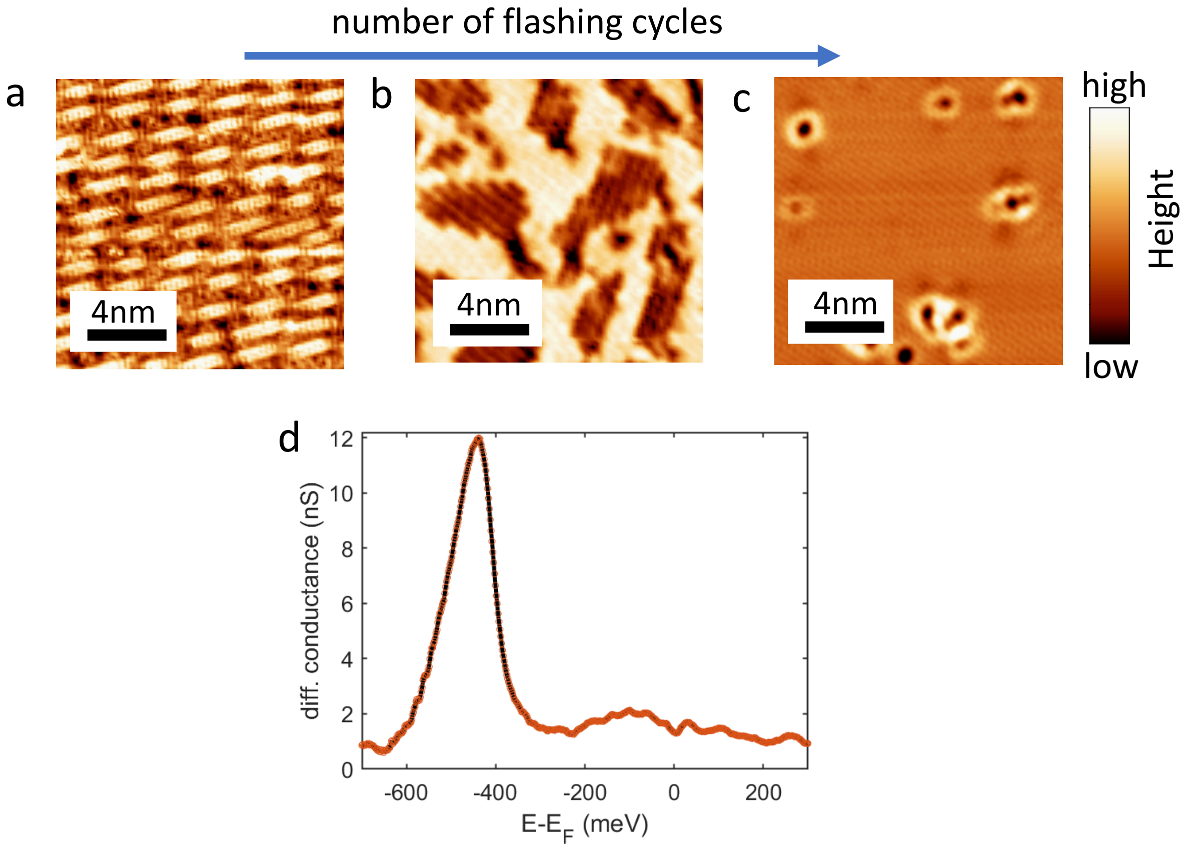

Supplementary Figure 1: Sample preparation. (a-c) Sample topography as a function of the number of flashing cycles. (a) Nb(110) surface after some flashing cycles at T=2200K, (b) Nb(110) surface after approximately 200 flashing cycles (T = 2300 K for 12 seconds followed by 3 minutes cooling time); (c) Nb(110) surface after approximately 300 flashes. (d) Scanning tunneling spectroscopy of the clean (110) surface. The high intensity peak visible at approximately 450 meV below the Fermi level corresponds to a surface resonance of d_{z^2} character [1].

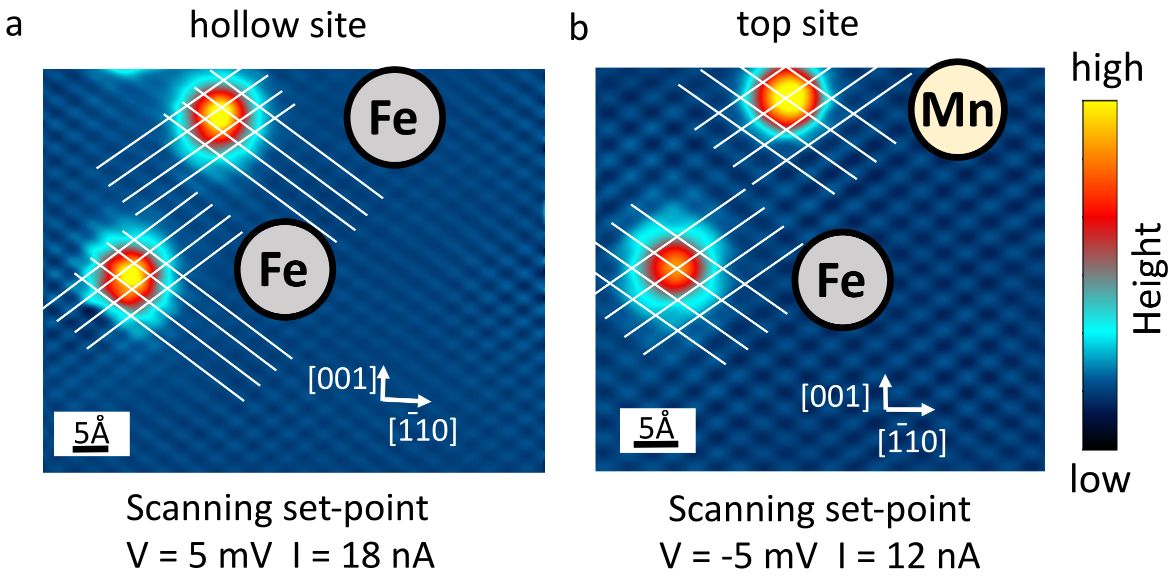

Supplementary Figure 2: Adsorption sites. All adatoms are characterized by a single and the same adsorption site, which is found to be the hollow site of the Nb(110) surface. The existence of a single adsorption site can be verified in two distinct way: (i) topographic imaging: as shown in the main text, Fig. 1 e, for each element, all adatoms have always the very same appearance on the surface; (ii) spectroscopic measurements: it is well-known that different adsorption sites result in very different hybridization strength, ultimately leading to distinct spectroscopic features. We analyzed several different adatoms for each element, and they all show the very same spectroscopic signatures, which is also found constant across different sample preparation (see Supplementary Note 4). The exact determination of the adsorption sites rely on atomically resolved images. Panel (a) show how adatoms are adsorbed, as expected, in the energetically favourable hollow site, which is the same adsorption site consistently used in our theoretical calculations (see Supplementary Note 9). However, depending on the tip used in the measurements and the set-point parameter, a corrugation reversal effect can be observed, as visualized in panel (b). This is in line with theoretical predictions suggesting strong corrugation reversal at (110) surfaces of *bcc* metals, with the effect expected to be particularly prominent especially for Nb [2].

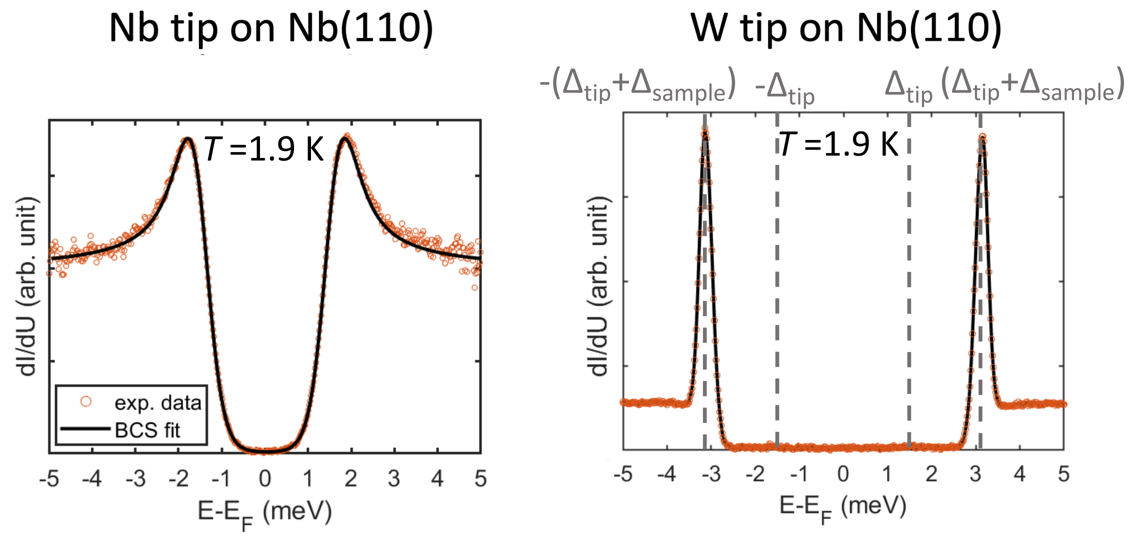

Supplementary Figure 3: Spectroscopy with superconducting tips. Scanning tunneling spectroscopy of the Nb(110) superconducting energy gap obtained with W (left panel) and Nb tip (right panel). When the superconducting Nb sample is measured with a W tip, the energy gap is smeared out, the energy resolution being limited by the width of the Fermi edge at the Fermi level. When the tip is superconducting, the energy resolution can be significantly improved, being only limited by the radiofrequency noise. In this case, tunneling between electron- and hole-peaks takes place at energies $\pm(\Delta_{\mathrm{tip}} + \Delta_{\mathrm{sample}})$, producing the sharp quasiparticle peaks visible in the spectra.

Supplementary Figure 4: Spectroscopic data for different adatoms. For each 3*d* element, the data are representative of measurements acquired on different sample preparations as well as by using different superconducting tips. For each 3*d* element, all adatoms always show the same behaviour. The energy position of the Shiba states strongly depend on adatom-substrate hybridization-dependent. The high reproducibility of the data confirms and further supports the existence of a single adsorption sites. Small differences in the absolute energy position of the Shiba peaks (orange curves) are related to the use of different Nb-coated tips. As described in Methods, they have been prepared by intending an electrochemically etched tungsten tip into the Nb(110) crystal, and they can show slight differences in their superconducting energy gap (black curves). However, the relative energy distance between Shiba peaks and single particle coherence peaks is tip-independent and it is consistently found to stay constant in all our measurements. See Supplementary Note 2.

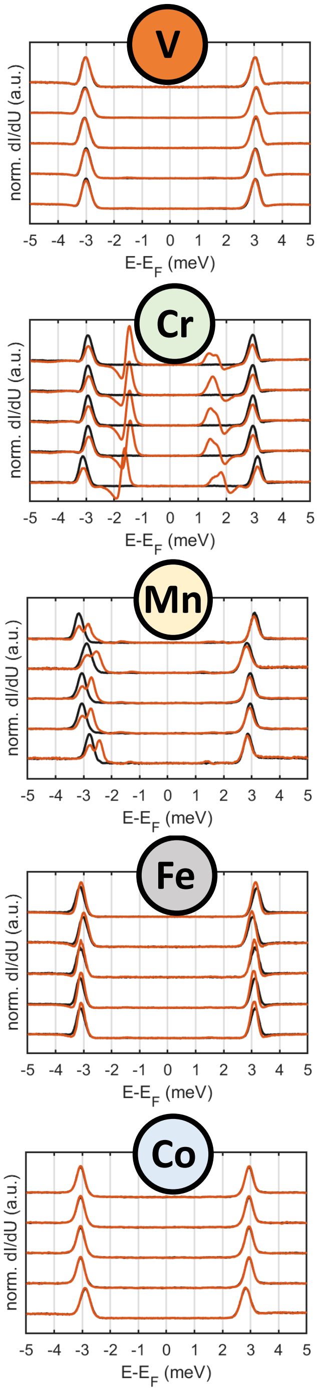

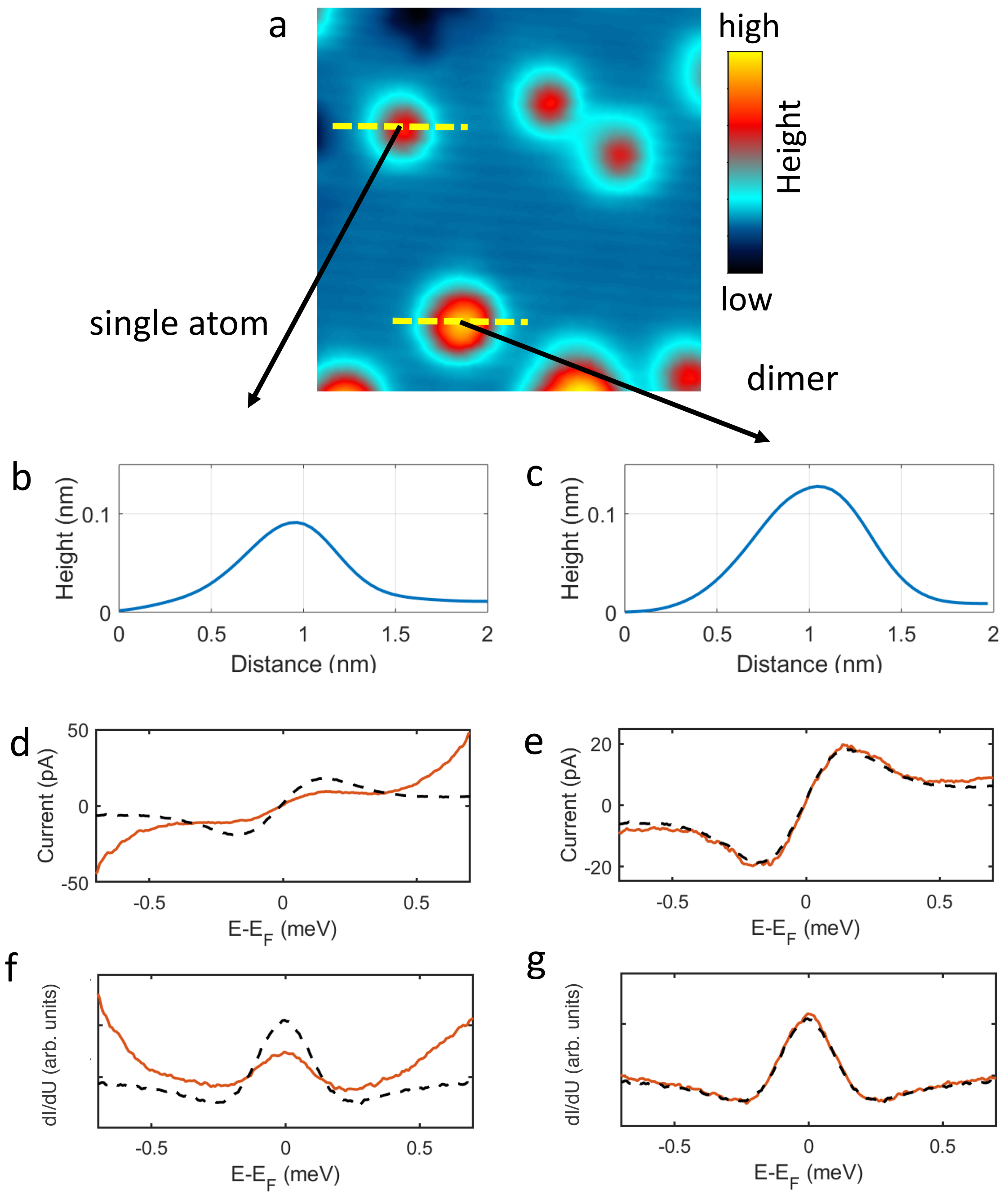

Supplementary Figure 5: Absence of tip height effects. (a) Topographic image showing one Cr adatom and one Cr dimer. The dimer has been created by tip-assisted atomic manipulation, (b) single Cr adatom and (c) Cr dimer apparent heights. (d,e) $I - V$ Josephson curves and respective (f,g) differential conductance for adatom and dimer, respectively. The Cr dimer is higher than the adatom. However, not any reduction compared to the bare Nb substrate is detected when positioning the tip on top of the dimer. This result is consistent with its antiferromagnetic ground state (S=0), ruling out tip-height artifacts.

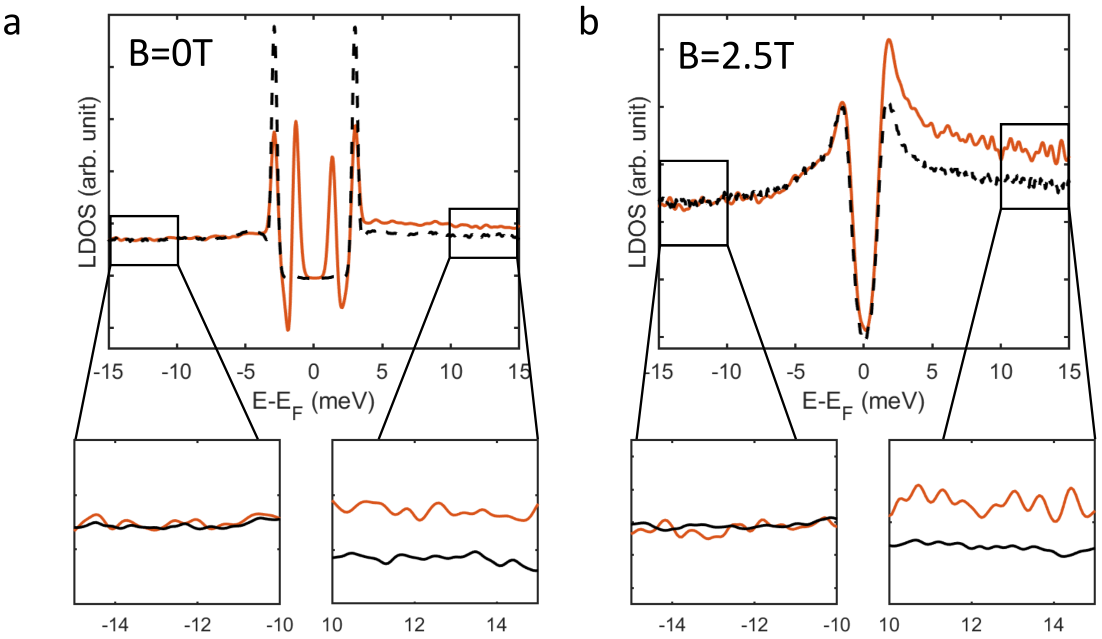

Supplementary Figure 6: Magnetic field-dependent spectroscopic data. The high intensity of the Cr spin-excitation makes possible to clearly visualize its coexistence when the superconducting state, as shown in (a). The emergence of a step function stays well visible when the Nb(110) substrate is turned in a normal metallic regime by applying a strong out-of-plane magnetic field (b). The residual gap visible in the spectrum corresponds to the tip superconducting gap, whose superconducting cluster at the apex is characterized by a much higher critical field than the sample due to its finite size.

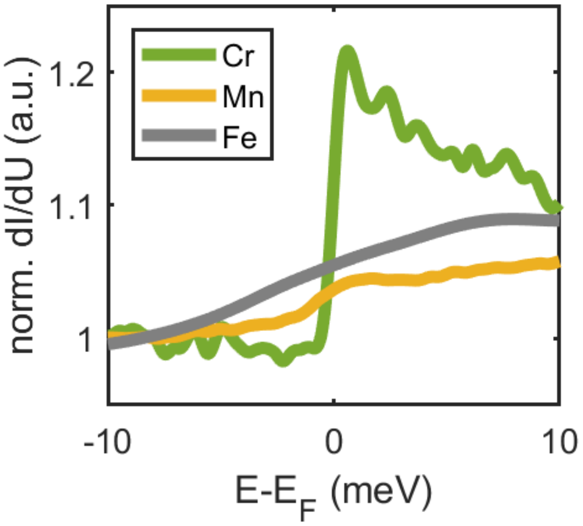

Supplementary Figure 7: Overlap of Cr, Mn, and Fe spectroscopic data in the metallic regime. A clear step-like feature is visible for Cr, with an intensity which is significantly stronger than those observed for the other atomic species.

Adatom	Nonmagnetic	Magnetic
Sc	8.2%	/
Ti	21.6%	20.5%
V	29.6%	22.5%
Cr	32.7%	17.2%
Mn	32.5%	18.2%
Fe	31.2%	28.6%
Co	28.8%	/
Ni	25.0%	/
Cu	17.6%	/
Zn	12.7%	/

Supplementary Table 1: Atomic relaxations of magnetic and nonmagnetic adatoms on Nb(110). Results obtained assuming the hollow site configuration illustrated in Supplementary Figure 8. All the adatoms relax towards the surface with numbers are given with respect to the Nb bulk interlayer distance.

Supplementary Note 1: Ground state properties from ab-initio

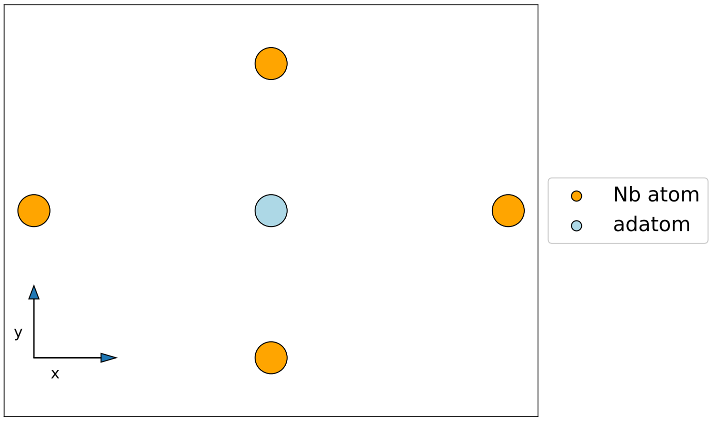

Supplementary Figure 8: Top view of the hollow site configuration. The adatoms represented by a blue circle are deposited on the bcc Nb(110) surface. The Nb atoms are shown as orange circles.

The geometrical optimization of the adatoms deposited on Nb was performed with the Quantum Espresso [3, 4] package. The lattice parameter of Nb is 6.25 a.u. and the vertical relaxations towards the surface obtained for the hollow site configuration (see Supplementary Figure 8) are summarized in Supplementary Table 1. The values are given with respect to the Nb bulk interlayer distance. One notices that V is relaxed by 22% and is predicted to be magnetic. We note that the corrugation measured experimentally indicates that V relaxes even more.

Based on the full-electron relativistic Korringa-Kohn-Rostoker (KKR) Green function method, we extracted the magnetic anisotropy energy (MAE) from either (i) the difference of the band energies obtained when the spin moment points along the in-plane x, y directions and the out-of-plane z direction (see Supplementary Figure 8) or (ii) the dynamical magnetic susceptibility [5]. Both methods lead to similar values and identical signs of the MAE, as presented in Supplementary Table 2. It was assumed that Cr and Mn are relaxed by 20% of the interlayer Nb distance towards the surface, while V, Fe and Co are relaxed by 30%. The bi-axial anisotropy characterizing the Nb bcc(110) surface is inherited by the impurities. In contrast to Mn and Fe, Cr prefers to have its spin moment pointing along the x-direction in the surface plane. The

	Band energy differences		Susceptibility	
Adatom	$E_z - E_x$	$E_z - E_y$	$E_z - E_x$	$E_z - E_y$
V	0.17	0.12	0.14	0.09
Cr	0.26	0.23	0.22	0.17
Mn	-0.77	-0.32	-0.90	-0.17
Fe	-0.84	-0.81	-0.86	-0.72

Supplementary Table 2: Magnetic anisotropy energies of adatoms. The values given in meV are obtained either from band energy differences or from the dynamical magnetic susceptibility. The calculations are based on the full-electron relativistic Korringa-Kohn-Rostoker Green function method. Positive (negative) values indicate an in-plane (out-of-plane) orientation of the spin moments. Owing to the (110) surface of bcc Nb, the in-plane directions along x and y directions (see Supplementary Figure 8) are not equivalent.

	Adatom	Nb (x-axis)	Nb (y-axis)	Cluster
V	1.20	0.14	-0.02	1.41
Cr	3.53	-0.14	-0.13	3.32
Mn	3.61	-0.38	-0.14	3.00
Fe	1.98	-0.32	-0.08	1.51

Supplementary Table 3: Spin magnetic moments. The values in μ_B are listed for the adatoms, the 2 nearest Nb atoms along each direction and the entire embedding cluster used in the ab-initio simulations.

in-plane MAE is more isotropic for Cr and Fe than for Mn.

As shown in Supplementary Table 3, the spin moments follow an inverse parabolic behavior as function of the atomic number of the adatoms with a maximum at half filling of the d-states. The neighboring Nb atoms carry a sizeable spin moment, which couples antiferromagnetically to the adatom moment (except the V neighbor along the x-axis). The closest Nb atoms, lying along the x-axis, have the largest spin moment. As mentioned in the main text, the total spin moment of the adatom-substrate complex can experience a strong modification because of the polarization cloud induced in the substrate. Supplementary Table 4 lists the orbital-decomposed spin and orbital moments of the adatoms. As expected, most of the moments are carried by the d-orbitals.

Supplementary Note 2: Adatom-substrate interaction

The calculation of the hybridization function is obtained by inverting the Green function of the adatom.

$$\mathcal{H} = E - G^{-1}(E), \tag{1}$$

Adatom	Spin moment				Orbital moment			
	**Total**	s	p	d	**Total**	s	p	d
V	**1.197**	0.035	0.013	1.149	**-0.026**	0.000	0.000	-0.026
Cr	**3.529**	0.074	0.005	3.452	**-0.029**	0.000	-0.001	-0.028
Mn	**3.606**	0.066	0.004	3.540	**0.020**	0.000	-0.001	0.021
Fe	**1.984**	0.036	-0.006	1.958	**0.083**	0.000	-0.001	0.085

Supplementary Table 4: Spin and orbital magnetic moments. Total and orbital-decomposed spin and orbital magnetic moments in μ_B of the adatoms.

Adatom	E_d	U	Γ	λ
V	0.322	0.433	-1.114	-0.004
Cr	-0.092	1.333	-0.895	0.010
Mn	-0.981	1.546	-0.885	0.013
Fe	-1.009	0.884	-0.765	0.006
Co	-0.996	0.000	-0.644	0.009

Supplementary Table 5: Ab-initio parameters for the onsite hamiltonian of the adatoms. The parameters, in eV, characterize the V, Cr, Mn, Fe and Co adatoms deposited on Nb(110) surface.

for which the local density of states (LDOS) of the adatom is obtained from the trace over the angular and spin momenta:

$$n(E) = -\frac{1}{\pi}\mathrm{Tr}_{Ls}\,\mathrm{Im}\,G(E) = -\frac{1}{\pi}\mathrm{Tr}_{Ls}\frac{1}{E-\mathcal{H}}. \quad (2)$$

After inverting the Green function, the resulting effective Hamiltonian hosts various terms such as the hybridization strength, the crystal field, and the strength of the spin-orbit coupling. The on-site hamiltonian of the adatom is given by

$$\mathcal{H} = \sum_{mm'}\sum_{ss'}\big(E_d\,\delta_{mm'}\delta_{ss'} + U\,\mathbf{e}\cdot\boldsymbol{\sigma}_{ss'}\delta_{mm'} + \lambda\,\mathbf{L}_{mm'}\cdot\boldsymbol{\sigma}_{ss'} + \Delta^{(\mathrm{re})}_{mm'}\delta_{ss'} + \mathrm{i}\Gamma\,\delta_{mm'}\delta_{ss'} + \mathrm{i}\Delta^{(\mathrm{im})}_{mm'}\delta_{ss'}\big)c^\dagger_{ims}c_{im's'} \quad , \quad (3)$$

where E_d is the average energy of the d-orbitals with respect to the Fermi energy, $2U$ represents the exchange splitting of the magnetic moment pointing along $\mathbf{e}$, $\boldsymbol{\sigma} = (\sigma_x, \sigma_y, \sigma_z)$ is the vector of Pauli matrices, λ is the strength of the local spin-orbit coupling, $\mathbf{L}$ is the local orbital angular momentum operator, $\Delta^{(\mathrm{re})}$ is an orbital dependent energy shift corresponding to the crystal field splitting, and Γ and $\Delta^{(\mathrm{im})}$ are non-hermitian contributions that result from the hybridization with the substrate. The different parameters are listed in Supplementary Tables 5, 6 and 7. The energy of the d-orbitals, E_d, lies below the Fermi energy for Cr, Mn, Fe and Co adatoms while it lies above the Fermi energy for V. The one of Mn, Fe and Co are shifted by about 1 eV from that of Cr and V. U, defining the exchange splitting, reaches a maximum for Mn, while Γ decreases monotically when increasing the atomic number of the $3d$ adatoms, as expected. The trends followed by Γ and $\Delta^{(\mathrm{im})}_{mm'}$ are listed in Supplementary Tables 6 and 7. We notice that d_{z^2} and d_{x-y^2} hybridize giving rise to two states, each being predominantly d_{z^2}- or d_{x-y^2}-like. The eigenvalues resulting from the diagonalization of the Hybridization function are plotted in Supplementary Figure 9, where one notices the same trend followed by Γ. Interestingly, the d_{x-y^2}-like (d_{xz}-like) state is the one hybridizing less (most) with the substrate. The little "bump" experienced by the d_{x-y^2}-like when going from V to Cr, Mn and down to Fe originates from the different atomic relaxations assumed for the various adatoms.

Supplementary Note 3: Theoretical evaluation of the spectrum of Yu-Shiba-Rusinov bound states

By virtue of the Schrieffer-Wolff transformation [6], the impurity-substrate s–d interaction $\mathcal{I}_m$ can be written as

$$\mathcal{I}^\sigma_m = (V_m + \sigma J_m S), \quad (4)$$

with V_m and J_m being the non-magnetic and magnetic scattering contributions, respectively. $\sigma = \pm$ indicates the spin of the conducting electrons and m is the magnetic quantum number (see also the analysis in Ref. 7). Here, $J > 0$ corresponds to an antiferromagnetic coupling. Within this approximation, the energies of the YSR states can elegantly be cast into [8, 9]:

$$\frac{\epsilon_m}{\Delta} = \pm\cos\left(\delta^+_m - \delta^-_m\right), \quad (5)$$

$\Delta^{(\mathrm{im})}$ V	xy	yz	z^2	xz	x^2-y^2
xy	-0.601	-0.000	-0.000	0.000	0.000
yz	-0.000	0.467	0.000	0.000	0.000
z^2	-0.000	0.000	0.044	0.000	-0.205
xz	0.000	0.000	0.000	-0.391	-0.000
x^2-y^2	0.000	0.000	-0.205	-0.000	0.481
$\Delta^{(\mathrm{im})}$ Cr	xy	yz	z^2	xz	x^2-y^2
xy	-0.378	-0.000	0.000	-0.00	0.000
yz	-0.000	0.362	0.000	0.00	-0.000
z^2	0.000	0.000	-0.105	0.00	-0.184
xz	-0.000	0.000	0.000	-0.54	0.000
x^2-y^2	0.000	-0.000	-0.184	0.00	0.660
$\Delta^{(\mathrm{im})}$ Mn	xy	yz	z^2	xz	x^2-y^2
xy	-0.33	0.0	0.000	0.000	0.000
yz	0.00	0.3	-0.000	0.000	0.000
z^2	0.00	-0.0	-0.079	-0.000	-0.167
xz	0.00	0.0	-0.000	-0.487	-0.000
x^2-y^2	0.00	0.0	-0.167	-0.000	0.595
$\Delta^{(\mathrm{im})}$ Fe	xy	yz	z^2	xz	x^2-y^2
xy	-0.393	-0.000	-0.000	-0.000	0.000
yz	-0.000	0.294	-0.000	0.000	-0.000
z^2	-0.000	-0.000	0.020	-0.000	-0.145
xz	-0.000	0.000	-0.000	-0.269	-0.000
x^2-y^2	0.000	-0.000	-0.145	-0.000	0.348
$\Delta^{(\mathrm{re})}$ Co	xy	yz	z^2	xz	x^2-y^2
xy	-0.254	0.000	0.000	0.000	0.000
yz	0.000	-0.007	-0.000	0.000	-0.000
z^2	0.000	-0.000	0.362	0.000	-0.115
xz	0.000	0.000	0.000	0.067	-0.000
x^2-y^2	0.000	-0.000	-0.115	-0.000	-0.168

Supplementary Table 6: Orbital-dependent hybridization interactions $\mathbf{\Delta^{(im)}}$. The values in eV are listed for V, Cr, Mn, Fe and Co adatoms deposited on Nb(110) surface.

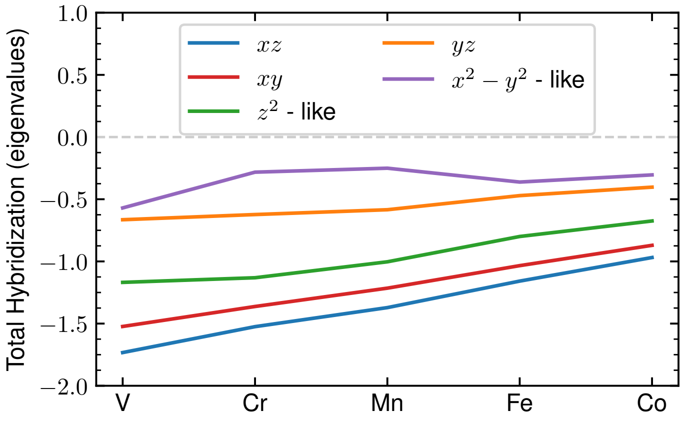

Supplementary Figure 9: Eigenvalues of the hybridization function. The plotted values given in eV correspond to the hybridization function of the d-orbitals of V, Cr, Mn, Fe and Co adatoms on Nb(110) surface.

$\Delta^{(\text{re})}$ V	xy	yz	z^2	xz	x^2-y^2
xy	-0.51	-0.000	0.000	0.000	0.000
yz	-0.00	0.119	0.000	0.000	-0.000
z^2	0.00	0.000	0.739	-0.000	-0.305
xz	0.00	0.000	-0.000	-0.091	0.000
x^2-y^2	0.00	-0.000	-0.305	0.000	-0.257
$\Delta^{(\text{re})}$ Cr	xy	yz	z^2	xz	x^2-y^2
xy	-0.075	0.000	0.000	-0.000	0.000
yz	0.000	-0.084	-0.000	0.000	0.000
z^2	0.000	-0.000	0.394	-0.000	-0.201
xz	-0.000	0.000	-0.000	-0.045	-0.000
x^2-y^2	0.000	0.000	-0.201	-0.000	-0.191
$\Delta^{(\text{re})}$ Mn	xy	yz	z^2	xz	x^2-y^2
xy	-0.067	-0.00	0.000	0.00	0.000
yz	-0.000	-0.13	0.000	0.00	-0.000
z^2	0.000	0.00	0.350	0.00	-0.164
xz	0.000	0.00	0.000	0.07	0.000
x^2-y^2	0.000	-0.00	-0.164	0.00	-0.223
$\Delta^{(\text{re})}$ Fe	xy	yz	z^2	xz	x^2-y^2
xy	-0.305	-0.000	0.000	-0.000	0.000
yz	-0.000	-0.004	0.000	0.000	0.000
z^2	0.000	0.000	0.439	-0.000	-0.142
xz	-0.000	0.000	-0.000	0.071	0.000
x^2-y^2	0.000	0.000	-0.142	0.000	-0.201
$\Delta^{(\text{im})}$ Co	xy	yz	z^2	xz	x^2-y^2
xy	-0.324	0.000	-0.000	0.000	0.000
yz	0.000	0.241	0.000	0.000	0.000
z^2	-0.000	0.000	0.014	0.000	-0.120
xz	0.000	0.000	0.000	-0.226	0.000
x^2-y^2	0.000	0.000	-0.120	0.000	0.296

Supplementary Table 7: Orbital-dependent crystal field interactions $\mathbf{\Delta}^{(\text{re})}$. The values in eV are listed for V, Cr, Mn, Fe and Co adatoms deposited on Nb(110) surface.

where the phase shifts are given by $\tan\delta_m^\sigma = \pi\rho\mathcal{I}_m^\sigma$.

The previous equation is fully equivalent to

$$\frac{\epsilon_m}{\Delta} = \pm\frac{1+{\beta_m}^2-{\alpha_m}^2}{\sqrt{[1+{\beta_m}^2-{\alpha_m}^2]^2+4{\alpha_m}^2}}, \tag{6}$$

which accounts for non-magnetic scattering effects, with $\beta_m = \pi\rho V_m$ and $\alpha_m = \pi\rho J_m S$. For a pure magnetic interaction, we recover the usual form

$$\frac{\epsilon_m}{\Delta} = \frac{1-{\alpha_m}^2}{1+{\alpha_m}^2},, \tag{7}$$

while a pure non-magnetic interaction gives $\frac{\epsilon_m}{\Delta} = 1$.

As mentioned in the main text, we map the scattering phase-shifts and the YSR energies from the interactions obtained from ab-initio as

$$\beta_m = \pi\rho V_m = -\frac{\Gamma_m E_m}{(E_m+U)(E_m-U)}; \quad \alpha_m = \pi\rho J_m S = \frac{\Gamma_m U}{(E_m+U)(E_m-U)}, \tag{8}$$

with E_m and Γ_m obtained after diagonalizing the hybridization function discussed in the previous section (see also Supplement of Ref. 7). The mapping was originally derived assuming an atom with a single electron occupying a given orbital hybridizing weakly with the electronic bath, which is characterized by a constant local density of states [6].

The theoretically-predicted energy position for the YSR states of Cr and Mn are summarized in Supplementary Table 8, also included in the main text. For both elements, the $d_{x^2-y^2}$- and d_{xy}-derived YSR states are located close to the edge and at the middle of the superconducting gap, respectively. This directly follows from the weak adatom-substrate antiferromagnetic exchange interaction, $\alpha_{d_{x^2-y^2}}$ listed in Supplementary Table 8. The d_{xy}-YSR state is the closest to the middle of the gap for both elements, which is originating from the large magnitude of $\alpha_{d_{xy}}$. Remarkably, the non-magnetic scattering, $\beta_{d_{xy}}$, is substantially large for Mn (0.978) adjusting thereby the impact of $\alpha_{d_{xy}}$ (1.447), which is a factor $\sim$50% larger than that of Cr (1.047). This is explained by the fact that $\frac{\beta_m}{\alpha_m} = \frac{E_m}{U}$ is enhanced if the energy of the d-orbital is pushed away from the Fermi energy. We note that this ratio for Mn is one order of magnitude larger than for Cr due to the $\sim$1 eV difference between their respective d-orbitals. For Cr, the energy of the YSR states of d_{yz} symmetry is predicted to be larger than that of the d_{z^2}. This is expected for Cr owing to $\alpha_{d_{yz}}$ (0.453) being weaker than $\alpha_{d_{z^2}}$ (0.795). The accompanying β does not modify substantially the position of the states, in contrast to those of Mn. There, α is similar for both states, but it is the non-magnetic part, β, that distinguishes the observed features. The latter has a larger binding energy for Mn than in Cr and presents a highly intense signal, since the d_{z^2} orbital can be more effectively measured by STM due to its larger extension into the vacuum. The extremely large adatom-substrate interactions characterizing V and Fe, induced by the LDOS resonance located at the Fermi energy, prohibit the use of our theoretical approach owing to the various assumptions and approximations.

In contrast to Fe, all the magnetic interactions of Cr and Mn are antiferromagnetic, see Supplementary Table 8. Overall, the interactions corresponding to the various orbitals are not purely magnetic and, as discussed earlier, they have a dramatic impact on the position of the YSR energies. While the d_{z^2} magnetic interaction is similar for Cr and Mn, about 0.7, the non-magnetic part changes by a factor of 2 (0.14 for Cr and 0.33 for Mn), which gives rise to the observed energy shift between the corresponding YSR energies. Thus, the large magnitude of the non-magnetic scattering induces the unusually huge electron-hole asymmetries observed experimentally for states close to the middle of the gap.

$\lvert\frac{\epsilon}{\Delta}\rvert$	xy	yz	z^2	xz	x^2-y^2
Cr	0.038	0.661	0.236	0.240	0.855
Mn	0.047	0.462	0.343	0.157	0.731

α_m	xy	yz	z^2	xz	x^2-y^2
V	28.530	2.257	-0.809	4.210	2.074
Cr	1.047	0.453	0.795	1.288	0.280
Mn	1.447	0.711	0.750	1.580	0.426
Fe	-13.191	-3.846	1.709	17.786	. -1.526
Co	0.000	0.000	0.000	0.000	0.000

β_m	xy	yz	z^2	xz	x^2-y^2
V	26.278	-1.344	1.507	-0.750	0.606
Cr	0.129	0.057	-0.140	0.146	0.044
Mn	0.978	0.496	0.335	0.996	0.306
Fe	-13.608	-4.043	1.336	17.258	-1.637
Co	0.804	0.401	1.033	1.299	0.248

Supplementary Table 8: Parameters describing the Yu-Shiba-Rusinov states. Orbital-dependent Yu-Shiba-Rusinov energies and non-magnetic and magnetic adatom-substrate interactions.

Supplementary Note 4: Spin-excitation properties obtained from time-dependent density functional theory

The various components of the dynamical spin susceptibilities calculated from time-dependent density functional theory (TD-DFT) are plotted in Supplementary Figure 10. The relevant susceptibility is χ^{+-} (or χ^{-+}), which measures the probably of reducing the spin of the adatoms by $\hbar$. Its imaginary part gives the density of spin-excitations. The position of the peak of the susceptibility correlates with the strength of the MAE, defining the energy barrier that the spin has to overcome to get excited. The broadening of the resonances is dictated by the electron-hole excitations which are approximately proportional to the product of opposite-spins density of states of the adatom at the Fermi energy.

Supplementary Note 5: Self-energy and renormalized density of state of the adatoms

In the main text, we have shown the renormalized density of states of the adatoms because of the presence of the spin-excitations. The scheme is described in great details in Refs. [10, 11, 5, 12, 13]. The theoretical scheme describing how spin-orbit coupling is incorportated can be found in Ref. [13].

The Green function is calculated from the Dyson equation

$$g(E) = \frac{G(E)}{1 - G(E)\Sigma(E)}, \tag{9}$$

where Σ is the self-energy describing the interaction of the electron and the spin excitations:

$$\begin{aligned}\Sigma^{\sigma\sigma'}(E) = -\frac{\mathcal{K}^2}{\pi}\sum_{ss'}\Bigg\{ &\int_0^{\infty} d\omega\, \mathrm{Im}\left[G^{ss'}(E+\omega)\,\chi^{\sigma s,s'\sigma'}(\omega)\right] \\ &- \int_0^{E_\mathrm{F}-\varepsilon} d\omega\, \mathrm{Im}\left[G^{ss'}(E+\omega)\right]\left[\chi^{\sigma s,s'\sigma'}(\omega)\right]^*\Bigg\} \quad ,\end{aligned} \tag{10}$$

where $\sigma\sigma'$ and ss' represent the spin blocks of respectively the self-energy and Green functions. The imaginary part of the self-energies, plotted in Supplementary Figure 11 are obtained from the imaginary part of

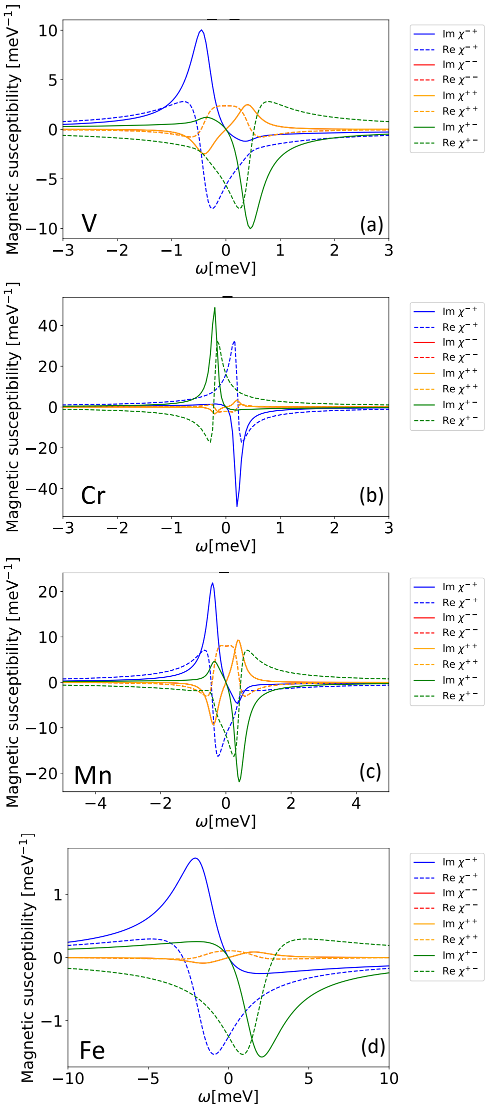

Supplementary Figure 10: Different components of the dynamical magnetic susceptibility. The imaginary and real parts of the susceptibility as function of energy are plotted for V, Cr, Mn and Fe adatoms on Nb(110).

the self-energy

$$\mathrm{Im}\,\Sigma^{\sigma\sigma}(E_{\mathrm{F}}+V) = -\frac{\mathcal{K}^2}{\pi}\int_0^{-V} d\omega\,\mathrm{Im}\left[G^{\bar{\sigma}\bar{\sigma}}(E_{\mathrm{F}}+V+\omega)\right]\mathrm{Im}\left[\chi^{\sigma\bar{\sigma},\bar{\sigma}\sigma}(\omega)\right]. \tag{11}$$

The latter equation indicates that $\mathrm{Im}\,\Sigma(E_{\mathrm{F}}+V)$ involves an integral of the susceptibility weighted by the density of states of the opposite spin channel. This explains why the imaginary part of the self energies have a shape of steps, which results from the integral over the resonances characterizing the density of spin-excitations. However the spin-asymmetry of the density of states of the adatoms can lead to large spin-asymmetries of the self-energies, as illustrated in the panels of Supplementary Figure 11. The self-energies renormalize the density of states, as shown in the main manuscript. Here we plot the spin-resolved density of states in the vacuum. Following the Tersoff-Hamann approximation [14], these quantities correspond to the

differential conductance measurable with scanning tunneling microscopy. The steps in the imaginary part of the self-energies translate into two spin-dependent dip-like features for Cr and Mn at positive and negative bias energies in the change of the density of states ($\Delta n = -\frac{1}{\pi}\mathrm{Im}\mathrm{Tr}_{Ls}(g-G)$), as shown in the right panels of Supplementary Figure 11. The overlap of the two features gives rise to the apparent step-like feature. For Fe, the majority-spin feature looks like a resonance, and is more prominent than the minority-spin broad step-like feature. The position of the two spin-resolved features is dictated by the strength of the magnetic anisotropy energy.

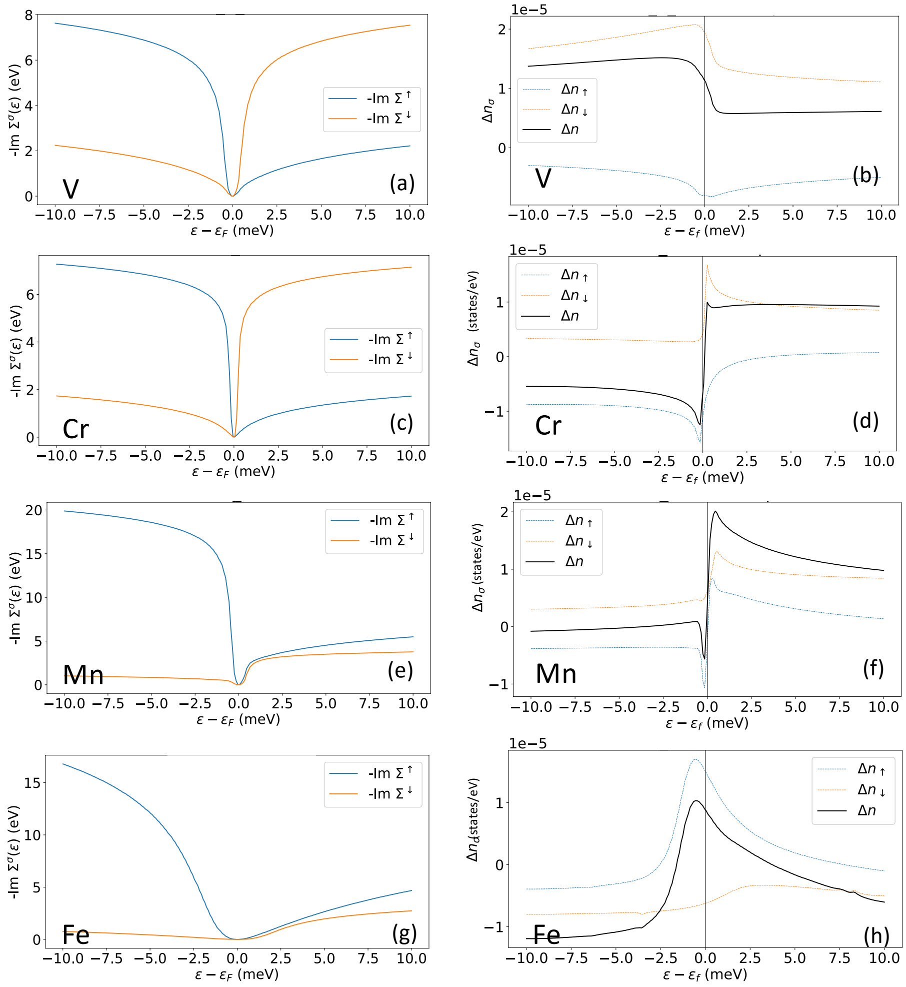

Supplementary Figure 11: Self-energies and LDOS for V, Cr, Mn and Fe adatoms. Spin-resolved self-energies (a,c,e,g) and change in the LDOS, Δn, induced by the presence of spin-excitations (b,d,f,h).

The step-like features of each spin channel can be described in terms of a Fano-Lorentzian curve, which can be parametrized by

$$n(E) = \mathrm{Im}\left(\exp(\mathrm{i}\Phi_q)\,\frac{A}{E - E_0 + \mathrm{i}\Gamma}\right) + n_0 \quad \text{with} \quad \Phi_q = 2\arctan(q) \quad , \tag{12}$$

where A is an amplitude, E_0 is the position of the resonance, Γ is the broadening, n_0 is a constant offset and Φ_q is a phase shift. To investigate the impact of the position of the resonance and the broadening, both driven by the magnetic anisotropy and an external magnetic field, we fit our theoretical renormalized density of states of the Fe adatom to a Fano-Lorentzian. Supplementary Figure 12a shows the fit for the

minority and the majority spin channel. The fitting parameters are shown in Supplementary Table **9**. The impact of an underestimated magnetic anisotropy is simulated by increasing both E_0 and Γ, which is shown in Supplementary Figure **12**b.

	E_0 (meV)	Γ (meV)	q	A (meV/MeV)	n_0 (1/MeV)
$\Delta n_\uparrow$	1.73	4.54	1.90	23.75	1.35
$\Delta n_\downarrow$	-0.95	1.57	3.08	32.24	2.39

Supplementary Table 9: Parameters fitting the zero-bias feature of Fe adatom. Values for the fit of a Fano-Lorentzian as parametrized in Supplementary Eq. (12) to the theoretical renormalized density of states of the minority and majority spin channels.

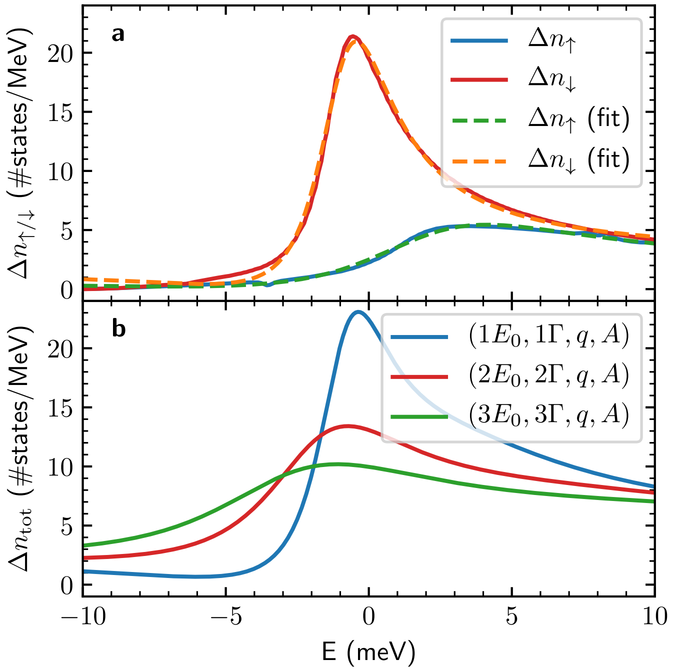

Supplementary Figure 12: Impact of the resonance position and its broadening on the local density of states. (a) Fit of the minority and majority spin channel of the theoretical renormalized density of states of the Fe adatom to a Fano-Lorentzian as parametrized in Supplementary Eq. (12). The fitting parameters are given in Supplementary Table **9**. (b) The total density of states as function of the resonance position E_0 and its broadening Γ. The impact of an underestimated magnetic anisotropy is simulated by increasing simultaneously E_0 and Γ. Shown are three different parameter sets with the original fitted parameters (blue curve) and a doubling of E_0 and Γ (red curve) as well as a tripling of E_0 and Γ (green curve).

References

[1] Odobesko, A. B. *et al.* Preparation and electronic properties of clean superconducting nb(110) surfaces. *Phys. Rev. B* **99**, 115437 (2019). URL https://link.aps.org/doi/10.1103/PhysRevB.99.115437.

[2] Heinze, S., Blügel, S., Pascal, R., Bode, M. & Wiesendanger, R. Prediction of bias-voltage-dependent corrugation reversal for stm images of bcc (110) surfaces: W(110), ta(110), and fe(110). *Phys. Rev. B* **58**, 16432–16445 (1998). URL https://link.aps.org/doi/10.1103/PhysRevB.58.16432.

[3] Giannozzi, P. *et al.* QUANTUM ESPRESSO: A modular and open-source software project for quantum simulations of materials. *Journal of Physics: Condensed Matter* **21**, 395502 (2009).

[4] Giannozzi, P. *et al.* Advanced capabilities for materials modelling with quantum ESPRESSO. *J. Phys.: Condens. Matter* **29**, 465901 (2017).

[5] dos Santos Dias, M., Schweflinghaus, B., Blügel, S. & Lounis, S. Relativistic dynamical spin excitations of magnetic adatoms. *Phys. Rev. B* **91**, 075405 (2015).

[6] Schrieffer, J. R. & Wolff, P. A. Relation between the anderson and kondo hamiltonians. *Phys. Rev.* **149**, 491–492 (1966). URL https://link.aps.org/doi/10.1103/PhysRev.149.491.

[7] Choi, D.-J. *et al.* Mapping the orbital structure of impurity bound states in a superconductor. *Nature Communications* **8**, 15175 (2017). URL https://doi.org/10.1038/ncomms15175.

[8] Rusinov, A. I. On the Theory of Gapless Superconductivity in Alloys Containing Paramagnetic Impurities. *JETP* **29**, 1101 (1969). URL https://doi.org/10.1143/PTP.40.435. https://academic.oup.com/ptp/article-pdf/40/3/435/5185550/40-3-435.pdf.

[9] Balatsky, A. V., Vekhter, I. & Zhu, J.-X. Impurity-induced states in conventional and unconventional superconductors. *Rev. Mod. Phys.* **78**, 373–433 (2006). URL https://link.aps.org/doi/10.1103/RevModPhys.78.373.

[10] Lounis, S., Costa, A. T., Muniz, R. B. & Mills, D. L. Dynamical Magnetic Excitations of Nanostructures from First Principles. *Phys. Rev. Lett.* **105**, 187205 (2010).

[11] Lounis, S., dos Santos Dias, M. & Schweflinghaus, B. Transverse dynamical magnetic susceptibilities from regular static density functional theory: Evaluation of damping and g shifts of spin excitations. *Phys. Rev. B* **91**, 104420 (2015).

[12] Schweflinghaus, B., dos Santos Dias, M., Costa, A. T. & Lounis, S. Renormalization of electron self-energies via their interaction with spin excitations: A first-principles investigation. *Phys. Rev. B* **89**, 235439 (2014).

[13] Bouaziz, J., Mendes Guimarães, F. S. & Lounis, S. A new view on the origin of zero-bias anomalies of co atoms atop noble metal surfaces. *Nature Communications* **11**, 6112 (2020). URL https://doi.org/10.1038/s41467-020-19746-1.

[14] Tersoff, J. & Hamann, D. R. Theory and Application for the Scanning Tunneling Microscope. *Phys. Rev. Lett.* **50**, 1998–2001 (1983).
